# Towards identifying dyslexia in Standard Indonesian: the development of a reading assessment battery

**DOI:** 10.1007/s11145-017-9748-y

**Published:** 2017-07-11

**Authors:** Bernard A. J. Jap, Elisabeth Borleffs, Ben A. M. Maassen

**Affiliations:** 1 International Doctorate for Experimental Approaches to Language and Brain (IDEALAB), University of Groningen, Groningen, The Netherlands; 2 University of Potsdam, Potsdam, Germany; 3 University of Newcastle, Newcastle upon Tyne, UK; 4 University of Trento, Trento, Italy; 5 Macquarie University, Sydney, Australia; 6 Science, Technology, and Society (STS) Research Group, Tarumanagara University, Jakarta, Indonesia; 7 Center for Language and Cognition Groningen (CLCG) and School of Behavioral and Cognitive Neurosciences (BCN), University of Groningen, P.O. Box 716, 9700 AS Groningen, The Netherlands

**Keywords:** Standard Indonesian, Dyslexia, Transparent orthography, Dyslexia assessment

## Abstract

With its transparent orthography, Standard Indonesian is spoken by over 160 million inhabitants and is the primary language of instruction in education and the government in Indonesia. An assessment battery of reading and reading-related skills was developed as a starting point for the diagnosis of dyslexia in beginner learners. Founded on the International Dyslexia Association’s definition of dyslexia, the test battery comprises nine empirically motivated reading and reading-related tasks assessing word reading, pseudoword reading, arithmetic, rapid automatized naming, phoneme deletion, forward and backward digit span, verbal fluency, orthographic choice (spelling), and writing. The test was validated by computing the relationships between the outcomes on the reading-skills and reading-related measures by means of correlation and factor analyses. External variables, i.e., school grades and teacher ratings of the reading and learning abilities of individual students, were also utilized to provide evidence of its construct validity. Four variables were found to be significantly related with reading-skill measures: phonological awareness, rapid naming, spelling, and digit span. The current study on reading development in Standard Indonesian confirms findings from other languages with transparent orthographies and suggests a test battery including preliminary norm scores for screening and assessment of elementary school children learning to read Standard Indonesian.

## Introduction

Although our understanding of dyslexia has grown in the past decades, in many non-English speaking parts of the world, the concept and study of dyslexia is still in its infancy (Lee, 2008). This also holds for Indonesia. With a population estimated to reach 258.7 million in 2016, Indonesia is the 4th most populous country on earth, ranking behind China, India, and the US. Figures vary, but numbers as high as 550 (Sneddon, 2003) and 731 (Frederick & Worden, 2011) have been mentioned for the different languages spoken in the Indonesian archipelago in the early 21st century. Standard Indonesian (SI) has become the language of schools, government, national print and electronic media, and of interethnic communication (Frederick & Worden, 2011). For about 23 million Indonesians nationwide SI is their primary language, while for over 140 million others SI is their second language (Lewis, Simons, & Fennig, 2013). Most of these first language SI-speakers live in larger cities.

Dyslexia occurs in all languages (Shaywitz, Morris, & Shaywitz, 2008), even though the consistency in which phonology is represented in the orthography varies and has a major effect in reading development (Ziegler & Goswami, 2005). The SI language script has a high degree of orthographic transparency with an almost one-to-one letter-to-sound correspondence (Winskel & Widjaja, 2007). Some cross-cultural work suggests universality in the neurocognitive and neurobiological causes of dyslexia (Peterson & Pennington, 2012). Based on previous studies conducted in other languages with transparent orthographies (e.g., Lyytinen, Erskine, Kujala, Ojanen, & Richardson, 2009; Trenta, Benassi, Di Filippo, Pontillo, & Zoccolotti, 2013; Van der Leij et al., 2013), it can be assumed that reading difficulties and dyslexia will also occur among the many Indonesians who (learn to) read SI. However, for those who are experiencing the negative consequences of these problems on their cognitive development, school motivation, well-being, and self-esteem (Lovio, Halttunen, Lyytinen, Näätänen, & Kujala, 2012), reading acquisition in SI has, unfortunately, not been studied widely and also, to the best of our knowledge, no standardized measures of reading skills and reading-related cognitive abilities have thus far been developed to diagnose dyslexia.

The development of a reading assessment battery is a crucial first step in the management of reading problems in Indonesia as knowledge and awareness of dyslexia in Indonesia are dependent on the accurate identification and treatment of individuals with or at risk of dyslexia in SI. The aim of the present study accordingly was to compose a comprehensive test battery to facilitate the assessment of reading acquisition and an early detection of reading difficulties in readers of SI. Such a test battery needs to comprise instruments to assess reading and spelling skills, as well as tools to evaluate cognitive functions known to be related to these skills.

### The orthography of Standard Indonesian

Standard Indonesian is a member of the Western Malayo-Polynesian subdivision of the Austronesian language family. Monolingual speakers of SI are relatively few in number; most Indonesians have first learned to speak regional dialects and only acquire SI through formal education.

The Indonesian alphabet consists of 26 letters that correspond to the English alphabet. Since the introduction of the Enhanced Indonesian Spelling System (EYD) in 1972, SI features a highly transparent orthography (formally, not considering local dialects) with all but one grapheme having a one-to-one grapheme-to-phoneme correspondence. The only grapheme with two possible phonological counterparts is the ‘e’, which either represents the schwa /ə/ or the /e/. Additionally, there are several digraphs (‘gh’, ‘kh’, ‘ng’, ‘ny’, ‘sy’). Indonesian has very few consonant clusters, only three diphthongs ‘ai’, ‘au’, ‘oi’, and six vowels /i/, /e/, /a/, /ə/, /o/, and /u/ (Moeliono & Dardjowidjojo, 1988). SI is a zero-marking language (Nichols & Bickel, 2013) without case or gender markers.

The syllable is a salient unit in SI, as multisyllabic forms make up the majority of words, rendering monosyllabic words uncommon (Winskel & Widjaja, 2007). Moreover, the syllable structures are simple and have clear boundaries, most frequently, V, VC, CV, CVC, and CVV (C = consonant; V = vowel; Prentice, 1987). More complex syllable structures and consonant clusters do exist but mainly through loanwords. Syllabic stress is regular and mostly falls on the penultimate or the final syllable (Gomez & Reason, 2002).

Indonesian possesses a rich transparent system of morphemes and affixations, with about 25 derivational affixes (Prentice, 1987). Nonaffixed forms are common in colloquial (spoken) Indonesian. The affixes have at least one semantic function and differ depending on the word class of the stem (Winskel & Widjaja, 2007). The stem word *makan* (‘to eat’) for example, becomes *termakan* (‘to be eaten’), *makanan* (‘food’), *makani* (‘devour’), and *pemakan* (‘eater’). There are irregularities, however, in the spelling of some affixes, which depend on their context. As many instructions in schoolbooks are written using these forms, Indonesian children need to be able to cope with long words consisting of various affixes from an early age (Winskel & Widjaja, 2007).

The salience of the syllable in SI is reflected in the formal teaching method adopted, which primarily focuses on teaching children about correspondences between whole spoken and written syllables rather than about grapheme–phoneme correspondences (Winskel & Lee, 2013). Formal reading instruction typically proceeds along the following lines: first the alphabet is introduced and children are trained to memorize the names of letters. Next, they are instructed to combine consonants and vowels into syllables with a simple C and V pattern, such as b + a, b + i, b + u, b + e, and b + o, producing the syllables *ba*, *bi*, *bu*, *be*, and *bo*. After rehearsing these syllables, the children are asked to combine the syllables to form a word, such as i + bu to create the word *ibu* (mother) (Dewi, 2003).

### Conceptual framework of the test battery

To conceptualize reading assessment in SI, we relied on the International Dyslexia Association’s (IDA) definition of dyslexia (Lyon, Shaywitch, & Shaywitz, 2003). According to this definition, dyslexia is characterized by problems with accurate and/or fluent word recognition (i.e., identifying real words), and poor spelling or decoding abilities (reading aloud pseudowords). These difficulties are often “unexpected” in view of the child’s other cognitive abilities (i.e., typical general intelligence) and exist despite the provision of adequate formal classroom instruction. At the explanatory level, dyslexia typically results from a deficit in the phonological component of language (Lyon et al., 2003).

**Fig. 1:**
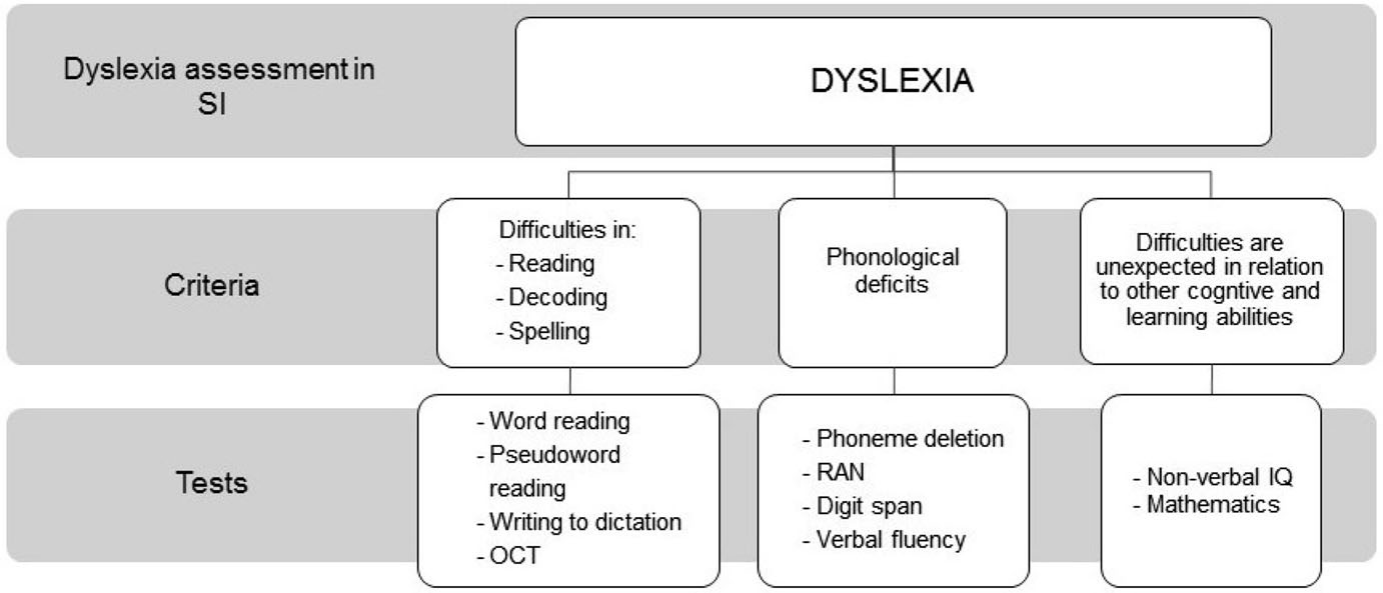
Conceptual framework of dyslexia

Following the IDA’s definition, we propose to identify dyslexia in SI based on three criteria: the presence of literacy difficulties that characterize dyslexia, a phonological component that underlies dyslexia, and an unexpectedness of these difficulties in relation to other cognitive and learning abilities. Building on this, the SI test battery then needs to employ a series of tasks to assess reading, decoding, and spelling skills. Moreover, as this initial assessment lacks specificity, measures of underlying phonological deficits need to be added to investigate other cognitive and learning abilities (Fig. 1).

### Assessment of compliance with dyslexia criteria

To gather evidence of literacy difficulties that most likely characterize dyslexia in SI, we assessed the reading, decoding, and spelling skills of young beginner readers using a single-word reading test, a pseudoword reading test, a writing-to-dictation task, and an orthographic choice (OCT) test. According to the simple view of reading, a model proposed by Gough and Tunmer (1986) and Hoover and Gough (1990), *decoding capacity* (defined as efficient word recognition) and *linguistic comprehension* (the ability to use information at the lexical or word level to achieve sentence and discourse interpretations) are both considered essential to reading success, while neither of the two skills is by itself sufficient. A key characteristic of dyslexia, which is word-level reading difficulties, is then explained by the lack of decoding skills (Gough & Tunmer, 1986; Rack, Snowling, & Olson, 1992), which becomes most apparent when words (e.g., pseudowords or non-words) cannot be read by sight (Snowling, 1995). Apart from lacking word-recognition and decoding skills, children with dyslexia often have spelling difficulties, which is not surprising as both theoretical and empirical studies argue that reading and spelling development are closely related (Ehri, 2005).

We included seven subtests in our battery to investigate potential phonological deficits underlying dyslexia in SI. Wagner and Torgesen (1987) distinguish three main types of phonological skills related to reading acquisition: phonological awareness (the sensitivity for and access to sounds in spoken words), rapid automatized naming (RAN; retrieval of phonological codes from long-term memory), and verbal short-term memory (phonological coding in short-term memory). These phonological abilities are assessed using a phoneme-deletion task, three different RAN tasks (digits, letters, colors), WISC (backward and forward) digit span, and a verbal-fluency task. Verbal fluency (e.g., operationalized as: Name as many words starting with /s/) has been shown to be relatively sensitive in distinguishing dyslexic readers with a phonological deficit from those with certain types of visual deficits, with the latter category performing within the normal range (Cohen, Morgan, Vaughn, Riccio, & Hall, 1999).

To gather evidence for a possible discrepancy between reading and spelling problems, and other cognitive or learning abilities, we utilized Raven’s “Coloured Progressive Matrices” (CPM; Raven & Court, 1962) to assess non-verbal IQ. In Indonesia, the CPM is one of the few standardized IQ tests for children and hence the most widely used. We furthermore investigated mathematical skills using the *Tempo Test Rekenen* [(Speeded Arithmetic Test); De Vos, 1992].

In the current study we aimed to address the following questions: (1) What are the cognitive factors indicative of typical reading ability in SI? (2) What is the typical profile of reading and reading-related cognitive skills for young beginner readers of SI? (3) Which variables distinguish typical young beginner readers from age peers with or at risk of developing dyslexia? And finally, (4) What are the optimal criteria for the categorization of reading difficulties in SI?

## Methods

### Participants

The participants were 75 first-grade students (44 boys; 31 girls) with a mean age of 6;4 (SD: 0.45; range 6;0–7;11) and 64 second-grade students (37 boys; 27 girls) with a mean age of 7;6 (SD: 0.52; range 7;0–9;8). All attended a Catholic private elementary school in West Jakarta, receiving education in SI, and came from middle socioeconomic backgrounds. Most were first language speakers of SI, though there was a small number of bilingual students who spoke a regional dialect at home (e.g., Javanese and Sundanese). It is worth noting that this language profile may not fit the majority of Indonesian speakers in the country, who usually acquire SI as a second language after the regional language spoken at home. The students were tested one month after the beginning of the second semester, with the first graders having received approximately 6 months and the second graders approximately 16 months of formal instruction.

### Materials and procedure

The tests were administered in two sessions, one group and one individual session. Individual testing times varied from 20 to 30 min, depending on the students’ abilities. The group session took place in the classroom and took approximately 1 h. The tests were administered by the first author with the assistance of psychologists. The words used for the reading tasks were drawn from the first-graders’ *Bahasa Indonesia* textbook to ensure that all children were familiar with the terms. Details of each task are exemplified below.

The group session comprised the following tests:

#### *Raven’s Coloured Progressive Matrices (CPM)*

The CPM (Raven & Court, 1962) was used to get an indication of the participants’ non-verbal intellectual capacities. Individual scores were compared with the average scores for that grade. All students completed the test and for those falling into the “poor reader” category, it was verified that they had above or equal-to-average intelligence according to the age-specific CPM norm scores; students scoring 13 or less (age 6;0) and 14 or less (age 7;0) were categorized as below average. The raw scores reflect the number of correct responses.

#### *Orthographic choice task (OCT)*

In this task the children were asked to identify the correct spelling (by underlining it) from three letter strings that were phonologically similar, as in *bisa* (correct; meaning: can), *bissa* (pseudoword), *byza* (pseudoword). A total of 20 sets of three letter strings were presented and the score was determined by the total number of correct answers.

#### *Writing to dictation*

Twenty words varying in phonological structure and length were presented orally in isolation and in sentence context. For example: *kaki* - *Manusia memiliki dua kaki.* (foot—Humans have two feet.). The students were instructed to write down the word using the correct spelling. The task score was based on the total number of correct answers.

#### *Tempo Test Rekenen (TTR)*

This arithmetic test (De Vos, 1992) assessed addition, subtraction, multiplication, and division. The first graders completed the first two sections (addition and subtraction), while the second graders additionally completed the multiplication section. The students were instructed to write down the answers as fast and as accurately as possible and, when the 1-min time limit had expired, they were told to proceed to the next section. The score was the average number of correct answers across the subtests administered.

The following tests were completed during the individual session:

#### *Word reading (reading)*

In this task, the student was presented with a list of 100 words taken from a first-semester grade-1 language textbook that were printed in columns on an A4-sized sheet. S(he) was asked to read aloud as many words as possible in 1 min, reading as fast and as accurately as possible. Included were words with different phonological structures and grapheme–phoneme mappings, with syllable lengths varying from 1 to 4. Examples of test items include *es* (ice), *mata* (eye), *baik* (good), *saudara* (sister), and *menyenangkan* (pleasant). The number of correctly pronounced words within the 1-min time limit was taken to reflect word-reading fluency.

#### *Pseudoword reading (decoding)*

This task used the words of the word-reading task albeit that several letters of each word have been modified. Examples are: *em*, *mita*, *boik*, *peudira*, and *kenyetangkin*. The children were instructed to read aloud as many pseudowords as possible in 1 min, where the number of correctly pronounced items was taken to indicate pseudoword reading fluency.

#### *Digit span*

The forward and backward digit-span tasks we used were adapted from the Dutch version of the WISC-R (Wechsler, 1974), of which the instructions were translated into SI. Students were asked to correctly repeat a sequence of digits immediately after presentation, in forward fashion during the first, and in backward fashion during the second task. Per trial two sequences with the same number of digits were presented. A trial was abandoned as soon as two errors were made on the same span length. Both digit-span scores were based on the number of correctly reproduced trials.

#### *Verbal fluency*

Students were asked to produce as many words starting with the phoneme /s/, excluding names of people and places. Verbal fluency was scored based on the number of words correctly produced in 1 min.

#### *Phoneme deletion*

The task consisted of 20 pseudowords that either had the initial, middle, or final phoneme omitted. The order of trials with different omissions was pseudo-randomized to prevent the children from being offered the same omission pattern more than twice in a row, which might cause confusion. For example, if the omission of an initial phoneme would be repeated multiple times, the participant might think the task only requires leaving out the first phoneme of each word. Moreover, to ensure that s(he) had heard the pseudoword correctly, the student was asked to repeat the pseudoword without the omission, just as it was orally presented by the experimenter (e.g., *piku* /pIkʊ/). Next, the student was instructed to repeat the pseudoword again, but now without the phoneme the experimenter provided. For example /pIkʊ/without the phoneme /ʊ/, resulting in the pseudoword /pIk/. Because the task could be challenging for the younger participants, the experimenter ensured comprehension of instructions in addition to providing multiple examples. The raw scores indicated the number of correct responses out of 20.

#### *Rapid automatized naming (RAN)*

As in the Van den Bos RAN subtasks (2003), the children were presented with capital letters, digits, or colors printed on a test sheet and asked to name these as fast and as accurately as possible. The time needed to name the total of 50 items constituted the test score. Afterwards, the score was converted into words per second (wps in Table 1), accounting for any skipped items.

**Table 1:** Descriptive statistics of the variables tested

	Grade 1 (n = 75)				Grade 2 (n = 64)			
	Mean	Min	Max	SD	Mean	Min	Max	SD
Reading fluency	63.03	39	100	14.73	76.95	54	99	11.73
Decoding fluency	44.75	20	99	14.74	54.14	31	91	12.00
Writing to dictation	18.79	6	20	2.08	19.39	14	20	1.00
OCT	17.88	0	20	2.67	19.23	16	20	0.99
TTR	11.68	0	24	4.72	16.45	8.67	32.67	4.44
Digit span forward	4.49	3	7	0.72	5.05	4	7	0.92
Digit span backward	2.80	2	5	0.75	3.05	2	6	0.86
Verbal fluency	5.52	0	14	3.40	6.77	0	14	3.10
Phoneme deletion	13.56	0	20	5.56	16.11	0	20	3.10
RAN digits—wps	1.41	0.49	2.09	0.34	1.76	1.11	2.52	0.30
RAN letters—wps	1.47	0.82	2.16	0.27	1.71	1.03	2.38	0.29
RAN colors—wps	0.86	0.36	1.64	0.22	0.98	0.50	1.79	0.20
CPM Score	25.91	11	35	5.13	27.59	11	35	5.13
Grades								
Language	83.16	66.00	95.00	7.02	83.10	65.70	93.30	7.58
Mathematics	82.56	62.00	94.00	7.19	81.52	54.60	93.40	7.71

Teacher ratings of individual difficulties	Mean	1	2	3	SD	Mean	1	2	3	SD
Learning in general	1.32	57	9	7	0.64	1.14	57	5	2	0.43
Reading	1.28	57	6	9	0.73	1.23	56	1	7	0.64
Writing	1.41	51	8	12	0.80	1.41	51	0	13	0.81
Mathematics	1.34	51	10	9	0.75	1.25	56	0	8	0.67

Teacher ratings: *1* no difficulties, *2* doubtful (not sure, it can be either 1 or 3); *3* noticeable difficulties. There are missing entries for teacher ratings in grade 1 as such: learning in general (2 missing); reading (3 missing); writing (4 missing); mathematics (5 missing)

#### *Teacher ratings*

For each grade, the homeroom teachers rated the students’ general learning, reading, writing, and arithmetic difficulties on a 3-point Likert scale, with the raw scores indicating the relevant numeric codes (1 = no difficulties, 2 = some difficulties/unable to determine, 3 = apparent difficulties).

#### *School grades*

Individual grades from the first-semester tests on SI (*Bahasa Indonesia)* and mathematics were made available to the researchers. The grades assigned were any number out of 100.

The analyses comprised: 1. variable descriptives; 2. correlation analysis; 3. factor analysis; 4. regression of cognitive and external variables on reading measures; 5. proposition of diagnostic cut-off values for each test and cross tabulation of poor performers to assess concordance of test outcomes; and 6. descriptives of the group of typical readers and the readers at risk of dyslexia. The correlation, factor, and regression analysis all contribute to the identification of reading-related cognitive factors. These factors build a cognitive profile of the tested individuals and distinguish at risk readers who display a dyslexic profile from typical readers, and from individuals who perform poorly on reading tasks for other reasons.

## Results

Table 1 lists the performance scores for the first and second graders on the 13 tests, in addition to their school grades and the teacher assessments of individual learning difficulties, presented as mean, minimal, and maximal values, and standard deviations. The reading and decoding fluency scores show the number of correctly produced words in 1 min. For writing to dictation, OCT, and phoneme deletion, correct answers out of 20 are given.

### Correlations

Interrelationships among 12 tests (excluding the CPM) and the school grades for language and mathematics were examined using a correlation matrix, where the non-shaded part of Table 2 shows the correlations for grade 1 and the shaded section the correlations for grade 2. Correlations were taken to be significant when *p* < 0.05.

**Table 2:**
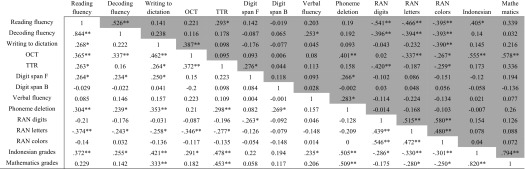
Correlation of variables for grade 1 (unshaded part, n = 75) and grade 2 (shaded part, n = 64)

** Correlation is significant at the 0.01 level (2-tailed)

* Correlation is significant at the 0.05 level (2-tailed)

Table 2 demonstrates that there are several variables that correlate significantly with reading and/or decoding fluency in both grades. In grade 1, reading fluency correlates significantly with decoding fluency, writing to dictation, OCT, arithmetic (TTR), digit span forward, phoneme deletion, RAN letters, and the language (SI) class grade. Similarly, decoding fluency correlates significantly with the aforementioned variables that correlated with reading fluency, with the exception of TTR and writing to dictation. Spelling (OCT) correlates with both reading measures, writing to dictation, TTR, RAN (letters), and language grades, while writing correlates with reading fluency, OCT, TTR, phoneme deletion, RAN (letters), and both language and math grades.

In grade 2, decoding fluency, TTR, all three RAN subtasks, and language grades correlate significantly with reading fluency, while reading fluency, verbal fluency and the three RAN tasks correlate significantly with decoding fluency. As for spelling (OCT), significant correlations can be observed for writing, phoneme deletion, RAN (letters and colors), language grades, and math grades, while writing to dictation correlates with OCT and RAN (colors).

The data also clearly shows that the correlations between RAN and reading or decoding fluency are more consistent and higher across the three RAN tasks for the second graders than they are for the first graders. Another marked difference is the significant correlation between the phoneme deletion task with reading or decoding fluency in grade 1 and the lack thereof in grade 2.

More reading-associated tasks correlated with actual reading measures in grade 1 than in grade 2. Spelling (OCT) scores correlated with both reading and decoding fluency in grade 1 but with neither in grade 2. The same can be observed for forward digit span, which correlated with the reading and decoding outcomes in grade 1 but not in grade 2.

In addition to the test battery tasks, the classroom language scores uncovered another distinction between the two groups in that they correlate with decoding fluency in grade 1 but not in grade 2. The language scores of the first graders moreover correlate with other analytical reading-related variables such as TTR, phoneme deletion, verbal fluency, and all three RAN tasks. Such a pattern is not found in grade 2, where the language score does correlate with the OCT scores and reading fluency, correlations that are also found in grade 1.

### Factor analysis results

A principal component analysis was conducted for the first graders, the results of which are shown in Table 3.

**Table 3:** Rotated component loadings for variables in grade 1

	Component		
	1	2	3
Reading fluency		0.907	
Decoding fluency		0.934	
Writing			0.728
OCT			0.665
TTR	0.338		0.550
Digit span-F		0.392	
Verbal fluency			0.519
Phoneme deletion			0.592
RAN digits	0.894		
RAN letters	0.758		
RAN colors	0.891		

Factor loadings <0.30 are suppressed

The analysis resulted in a three-factor solution, with the first factor being composed of the three RAN tasks and a moderate loading of arithmetic (TTR), which can be interpreted as the automaticity required to complete these tasks. The second factor is composed of word reading, pseudoword reading (decoding), and forward digit span, which, given their loadings, would earmark this factor as a reading component. The third factor is mainly composed of writing to dictation, OCT, arithmetic, verbal fluency, and phoneme deletion, and can hence be said to reflect writing, spelling, and reading-related skills.

The results of the principal component analysis for the second graders are shown in Table 4.

**Table 4:** Rotated component loadings for variables in grade 2

	Component		
	1	2	3
Reading fluency	0.742		
Decoding fluency	0.631		
Writing		0.335	
OCT		0.713	
TTR	0.482		0.405
Digit span-F		0.373	0.745
Verbal fluency		0.467	
Phoneme deletion		0.819	
RAN digits	0.879		
RAN letters	0.764		
RAN colors	0.761		

Factor loadings <0.30 are suppressed

The analysis yielded three components. The first component comprises two sets of measures, i.e., the two reading measures (word and pseudoword reading), and the arithmetic and RAN tasks. Successful performance of all these tasks requires automaticity and some degree of verbal skills. The second component is much like the third component for grade 1, comprising writing and spelling, and forward digit span, verbal fluency, and phoneme deletion (mostly reading-related verbal measures). The third factor includes arithmetic and forward digit span, again cognitive reading-related variables.

### Regression of cognitive and external variables on reading measures

A multiple regression analysis was conducted with the factor scores of grade 1 but excluding reading and decoding fluency, producing three discernible components (“Appendix 1”) to predict reading and decoding fluency. The model was a good fit for reading fluency [*F*(3,71) = 8.008, *p* < 0.001, *R* ^2^ = 0.253] and components 1 (*p* < 0.001) and 2 (*p* = 0.004) are both significant predictors. As for decoding fluency [*F*(3,71) = 4.091, *p* = 0.010, *R* ^2^ = 0.147], component 2 (*p* = 0.002) significantly predicts decoding. As can be seen in “Appendix 1”, component 2 is comprised of mainly writing, OCT, TTR, and phoneme deletion.

The same set of factors was applied to model reading and decoding fluency of grade 2. As with grade 1, the set of components (“Appendix 2”) formed a model that fitted for reading fluency [*F*(3,60) = 11.336, *p* < 0.001, *R* ^2^ = 0.362] and decoding fluency [*F*(3,60) = 5.922, *p* = 0.001, *R* ^2^ = 0.228]. One of the components, namely RAN/automaticity (component 1), significantly predicted reading (*p* = 0.000) and decoding fluency (*p* = 0.001).

In addition, multiple linear regression analyses were performed to predict reading and decoding fluency based on the external variables (teacher ratings and school grades). For the first graders, a significant regression equation was found for reading fluency [*F*(6,66) = 2.682, *p* = 0.022], with an *R* ^2^ of 0.196, with language grades being the only variable to significantly predict (*p* = 0.018) this dependent variable. The external variables did not significantly predict decoding fluency in grade 1 [*F*(6,66) = 1.609, *p* = 0.158, *R* ^*2*^ = 0.128]. Notably, as a predictor of decoding fluency in the first grade, the teachers’ assessment of the students’ writing difficulties was close to the significance threshold (*p* = 0.079).

In grade 2, the external variables were less prognostic: neither language and mathematics grades nor teacher ratings significantly predicted reading fluency [*F*(5,25) = 1.841, *p* = 0.141, *R* ^*2*^ = 0.269] or decoding fluency [*F*(5,25) = 0.409, *p* = 0.838, *R* ^*2*^ = 0.076].

### Cross tabulation and categorization of reading and decoding difficulties

Consistent with the main features of dyslexia (IDA: problems with accurate and/or fluent word recognition and poor spelling or decoding abilities), the number of poor readers, decoders and/or spellers in our sample were classified according to percentile scores. We were especially interested to see whether the children that were identified as poor readers were also poor decoders and/or spellers. For each test separately any diagnostic cut-off point would be arbitrary, but since percentiles can express substandard performance, we could propose a sensible combination of poor scores that could be indicative of reading difficulties, thereby putting the child in an at-risk category of poor literacy development. This method of classifying subgroups with lower reading performance percentiles as dyslexic has been used in numerous studies (Lovett, Steinbach, & Frijters, 2000; Torgesen, Wagner, Rashotte, Burgess, & Hecht, 1997). Using similar dyslexia criteria proposed for young Dutch language learners (Van der Leij et al., 2013) and based on the 10th, 20th, and 40th percentiles of the present sample, we suggest two at-risk categories for young beginner readers of SI. The cut-off values for these percentile criteria were calculated by using the means and *SD*s (with *Z*-critical values) for the total group of children (e.g., P10 = mean − 1.28 × *SD*).

The first at-risk category includes poor readers and/or decoders with the following scores:

- ≤P10 on reading (<45 for grade 1, <62 for grade 2) *and* ≤P40 on decoding (<42 for grade 1, <52 for grade 2).

*or*

(b) ≤P10 on decoding (<26 for grade 1, <39 for grade 2) *and* ≤ P40 on reading (<60 for grade 1, <75 for grade 2).

The second at-risk category includes poor spellers and/or writers that also have relatively poor reading and/or decoding skills, as reflected by the following scores:

- ≤P20 on reading (<51 for grade 1, <68 for grade 2) and/or decoding scores (<33 for grade 1, <45 for grade 2).

*and*

- ≤P10 on spelling (OCT; <15 for grade 1, <19 for grade 2) *and* ≤P40 on writing to dictation (<19 for grade 1, <20 for grade 2).

*or*

(b) ≤P10 on writing to dictations (<17 for grade 1, <19 for grade 2) *and* ≤P40 on spelling (OCT; <18 for grade 1, <20 for grade 2).

When these criteria are applied, there is an overlap between poor reading and decoding, and poor spelling. The results for each grade, indicating the classifications and numbers of students that were found to be at risk of dyslexia, are presented in Table 5.

**Table 5:** At-risk classifications and number of students per category per grade

	Category	Grade 1	Grade 2
Reading ≤10th and decoding ≤40th*	1a	9	4
Decoding ≤10th and reading ≤40th*	1b	2	2
Reading and decoding ≤10th	1a and 1b	2	3
Category 1 total		13	9
Spelling ≤10th and writing ≤40th**	2a	1	0
Writing ≤10th and spelling ≤40th**	2b	0	0
Spelling and writing ≤10th	2a and 2b	1	1
Category 2 total		2	1
Overlap Category 1 and 2		2	1
Total at risk of dyslexia		13	9

* Excluding ‘Category 1a and 1b’; ** Excluding ‘Category 2a and 2b’

As shown in Table 5, 13 first graders and nine second graders were found to have poor reading/writing/spelling skills, who are potentially at risk of developing dyslexia. All students who qualified for the poor spellers/writers criteria also fell into the poor readers/decoders group.

### Descriptives of typical and at-risk readers

We next compared the variables for the typical readers and those found to be at risk of dyslexia according to the criteria. Descriptive values and results of the group comparison are presented in Table 6. It is important to appreciate that while the at-risk group had lower scores on numerous variables tested, the group had average or above average non-verbal intelligence, as tested with the CPM.

**Table 6:** Descriptive statistics and *t* test results of typical readers and at-risk readers per grade

	Grade 1 (n = 75)										
	Typical (n = 62)				At-risk (n = 13)				Mean diff. (*t* test)		
	Mean	Min	Max	SD	Mean	Min	Max	SD	*t*	*df*	*p*
Reading fluency	67.29	48	100	12.42	42.69	39	50	3.52	7.05	73	<.001*
Decoding fluency	48.08	28	99	13.95	28.85	20	35	4.38	4.89	73	<.001*
Writing to dictation	19.02	13	20	1.52	17.69	6	20	3.66	2.14	73	0.036*
OCT	18.13	0	20	2.80	16.69	14	19	1.49	2.61	73	0.013*
TTR	12.03	0	24	4.99	10.00	3.5	14	2.59	1.42	73	0.16
Digit span forward	4.55	3	7	0.76	4.23	4	5	0.44	1.45	73	0.15
Digit span backward	2.79	2	5	0.77	2.85	2	4	0.69	−0.26	73	0.798
Verbal fluency	5.42	1	14	3.38	6.00	0	12	3.58	−0.54	73	0.599
Phoneme deletion	14.24	0	20	5.15	10.31	0	18	6.45	2.39	73	0.019*
RAN digits—wps	1.45	0.49	2.09	0.35	1.26	0.94	1.67	0.24	1.81	73	0.08
RAN letter—wps	1.52	0.94	2.16	0.26	1.26	0.82	1.47	0.18	4.19	73	<.001*
RAN color—wps	0.87	0.36	1.64	0.23	0.78	0.64	0.89	0.09	1.35	73	0.181
CPM score	26.05	11	35	5.09	25.23	14	32	5.50	0.49	73	0.628
Grades											
Language	83.79	66	95	7.16	80.00	68	88	5.49	2.06	71	0.052
Mathematics	82.90	62	94	7.48	80.83	68	88	5.39	1.13	71	0.271

Teacher Ratings of indiv. difficulties	Mean	1	2	3	SD	Mean	1	2	3	SD	*t*	*df*	*p*
Learning in general	1.25	50	7	4	0.57	1.67	7	2	3	0.89	−2.12	71	0.037*
Reading	1.23	52	3	6	0.64	1.54	5	3	3	1.05	−1.42	73	0.16
Writing	1.26	47	6	6	0.68	2.17	4	2	6	0.94	−3.94	71	<.001*
Mathematics	1.28	46	7	6	0.69	1.67	5	3	3	0.99	−1.66	71	0.101

	Grade 2 (n = 64)										
	Typical (n = 55)				At-risk (n = 9)				Mean diff. (*t* test)		
	Mean	Min	Max	SD	Mean	Min	Max	SD	*t*	*df*	*p*
Reading fluency	79.73	56	99	10.00	60.00	54	73	5.74	5.74	62	<.001*
Decoding fluency	56.60	40	91	10.78	39.11	31	51	7.34	6.15	62	<.001*
Writing to dictation	19.53	17	20	0.72	18.56	14	20	1.88	2.85	62	0.006*
OCT	19.36	16	20	0.95	18.44	17	19	0.88	2.87	62	0.015*
TTR	16.82	8.67	32.67	4.53	14.15	10.67	20	3.11	2.2	62	0.043*
Digit span forward	5.09	4	7	0.89	4.78	4	7	1.09	0.82	62	0.434
Digit span backward	3.06	2	6	0.89	3.00	2	4	0.71	0.21	62	0.84
Verbal fluency	6.93	0	14	3.07	5.78	2	12	3.31	0.98	62	0.351
Phoneme deletion	16.46	10	20	2.30	14.00	0	20	5.85	2.27	62	0.027*
RAN digits—wps	1.79	1.11	2.52	0.30	1.61	1.32	1.92	0.23	2.04	62	0.062
RAN letter—wps	1.74	1.03	2.38	0.29	1.52	1.28	1.92	0.20	2.88	62	0.012*
RAN color—wps	1.00	0.63	1.79	0.19	0.85	0.5	1.21	0.21	1.94	62	0.081
CPM score	27.67	11	35	5.20	27.11	18	33	5.01	0.31	62	0.76
Grades											
Language	84.10	65.8	93.3	7.01	73.80	65.7	80.1	7.37	2.409	29	0.023*
Mathematics	82.45	68.7	93.4	6.15	72.87	54.6	84.1	15.96	2.17	29	0.038*

Teacher Ratings of indiv. difficulties	Mean	1	2	3	SD	Mean	1	2	3	SD	*t*	*df*	*p*
Learning in general	1.09	51	3	1	0.35	1.44	6	2	1	0.73	−2.36	62	0.021*
Reading	1.15	51	0	4	0.52	1.78	5	1	3	0.97	−2.93	62	0.005*
Writing	1.22	49	0	6	0.63	2.56	2	0	7	0.88	−4.37	62	0.002*
Mathematics	1.18	50	0	5	0.58	1.67	6	0	3	1.00	−2.08	62	0.042*

Teacher ratings: *1* no difficulties, *2* doubtful (not sure, it can be either 1 or 3), *3* noticeable difficulties

* Mean difference is significant at the 0.05 level

**Fig. 2:**
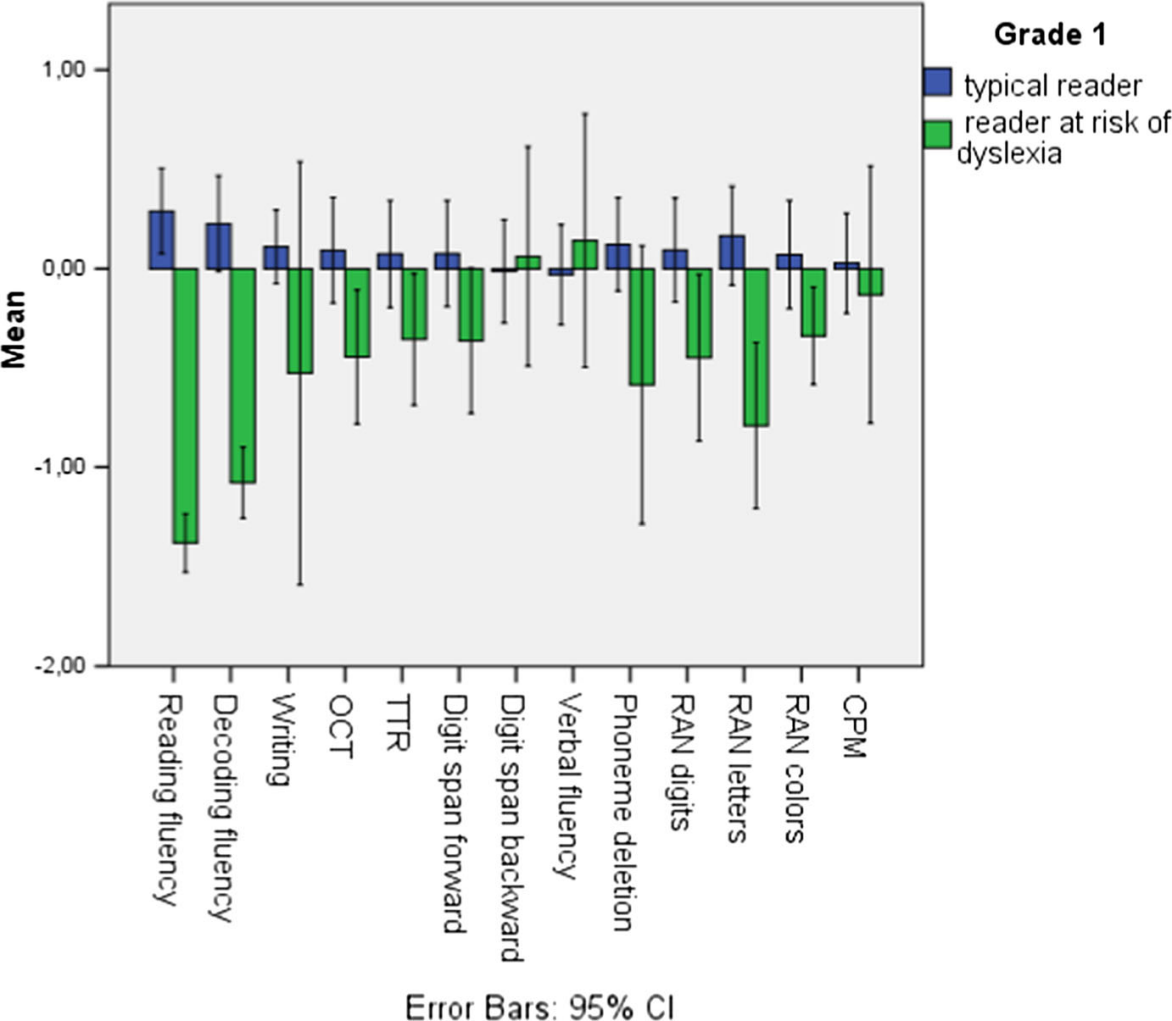
Z-scores of typical readers and at-risk readers in grade 1

**Fig. 3:**
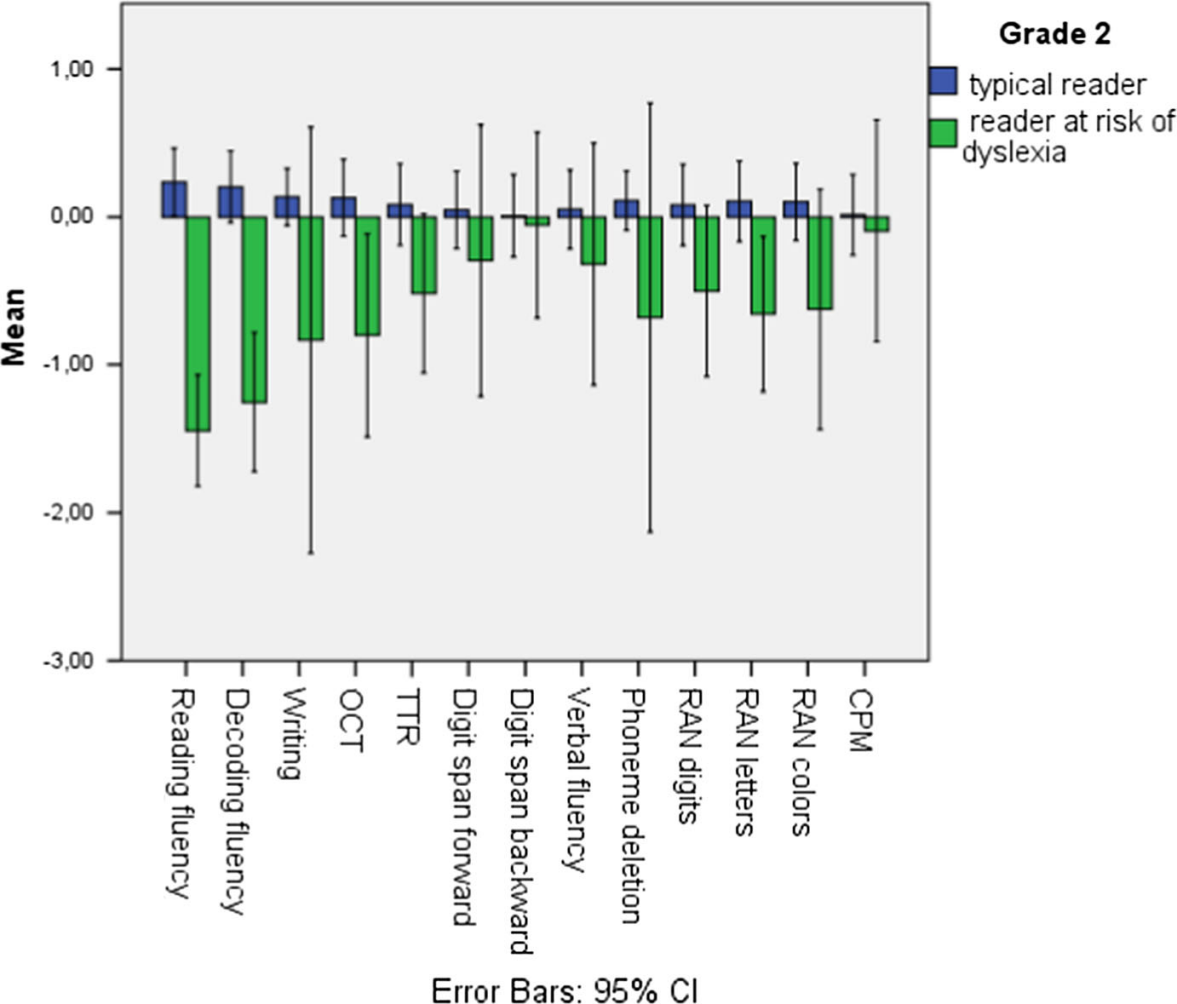
Z-scores of typical readers and at-risk readers in grade 2

Independent samples *t* tests were conducted to statistically evaluate the differences between the two groups per grade as shown in Table 6. Figures 2 and 3 display group differences per grade in a more graphic manner, by using z-scores calculated for each variable per grade.

Both grades show significant group variations in reading and decoding fluency (Table 6). This is unremarkable because reading and decoding fluency are part of the selection criteria. However, in grade 1, the at-risk group scored significantly lower on writing to dictation, and OCT; recall that only two out of the 13 children at risk based on reading and decoding, were also at risk based on spelling. In addition, the at-risk children scored significantly lower on phoneme deletion, and a RAN subtask (letters). Additionally, the grade-1 teachers rated the children in the at-risk group to have significantly more problems in learning in general and writing. In grade 2, the at-risk group had lower scores for writing, OCT (only one out of nine being at risk based on spelling), arithmetics (TTR), phoneme deletion, and a RAN subtask (letters). Moreover, similar to grade 1, the grade-2 teacher ratings for the at-risk group were significantly worse for general learning, reading, writing, and mathematics. To add to this, the school grades for language and mathematics of the grade-2 at-risk group were significantly lower. It is lastly important to note that the non-verbal IQ scores (CPM) were not significantly different between the groups across grades.

The analyses demonstrated that the at risk of dyslexia criteria we adopted were to some extent supported by the external variables (teacher ratings and school grades): the grades for the at-risk group also indicate that their school performance was poorer.

## Discussion

The aim of the present study was to develop a comprehensive test battery that would allow the assessment of reading acquisition and the early detection of reading difficulties in beginner learners of Standard Indonesian (SI). Based on the results attained by typical young readers, we further aimed to propose criteria to differentiate children with and without dyslexia and those at risk of developing the disorder.

Ziegler and Goswami (2005) argue that in alphabetic languages predictors of reading performance are relatively universal. However, the predictors’ precise weights may be modulated by the transparency of the orthography, as may also be the case for the indicators of dyslexia. SI orthography has a high degree of transparency with a close-to-perfect letter-to-sound correspondence (Winskel & Widjaja, 2007). Reading research has shown that slowed reading speed is the most marked problem in dyslexic readers in transparent orthographies (Tilanus, Segers, & Verhoeven, 2013; Wimmer, 1993) and that reading accuracy remains relatively unaffected following the very early stages of reading acquisition (Holopainen, Ahonen, & Lyytinen 2001; Landerl, Wimmer, & Frith, 1997; Tressoldi, Stella, & Faggella, 2001). We accordingly took reading and decoding fluency as the main components of the test battery, with speed and accuracy being measured as the number of correctly read words and pseudowords within 1 min.

To gather evidence of literacy difficulties that may characterize dyslexia in SI, we assessed reading, decoding, and spelling abilities, in addition to phonological skills and other cognitive aspects related to reading in beginner learners of SI. The results indicated that several of our tests correlated significantly with reading and decoding fluency. Most notably, the correlations between rapid automatized naming (RAN) and reading or decoding fluency were more consistent and higher across the three RAN tasks in the grade-2 than they were in the grade-1 readers. Moreover, whereas the phoneme deletion task correlated significantly with reading or decoding fluency in grade 1, this was not the case for grade 2. These findings agree with results from earlier research in languages with transparent orthographies and indicate a decreasing effect of phonological awareness on reading after starting school (formal instruction) when the basic decoding rules have been learned (e.g., De Jong & Van der Leij, 2002; Georgiou, Parrila, & Papadopoulos, 2008; Holopainen et al., 2001), whereas the importance of RAN over time appears to increase (De Jong & Van der Leij, 1999; Vaessen & Blomert, 2010). Overall, the correlations between reading and decoding fluency and other reading-related tasks were higher in grade 1 than in grade 2. The outcomes on the orthographic choice task (OCT) and writing to dictation, for example, correlated significantly with reading fluency (and with decoding fluency for OCT) in grade 1 but not in grade 2. Note that the results we obtained may have been influenced by the level of the task content, which for both tasks was based on grade-1 textbooks and therefore relatively easy for the second graders, which is also reflected in the high mean scores for these students on the two tasks (Table 1). We consequently propose to increase the complexity of these spelling tests for grade 2 and above. The principal component analysis we conducted resulted in a three-factor solution in both grades, including two separate components for reading and decoding, and for writing and spelling. Regarding the tasks mentioned under the ‘phonological deficit criterion’ in Fig. 1, the factor analysis in grade 1 yielded a separate RAN component, whereas in grade 2 the RAN tasks were incorporated in the reading and decoding component. In both grades, verbal fluency and phoneme deletion were part of the writing and spelling component.

The independent sample *t* tests showed significant differences between the typical and the at-risk readers for reading and decoding fluency as well as for phoneme deletion, writing to dictation, OCT, and RAN in both grades. Additionally, based on external measures, the at-risk second-graders performed more poorly on language and mathematics, and in both grades, teacher ratings pointed out some form of difficulties in writing, reading, mathematics, or studying in general in the at-risk group.

Based on our categorization criteria, 17.3% of the first graders and 14.1% of the second graders were found to be at risk of dyslexia. Depending on the definition, linguistic system, and the stringency of the criteria used, in western populations the prevalence of developmental dyslexia varies between 5 and 10%, and up to 17.5% for English speakers (Gilger, Pennington, & De Fries, 1991; Habib, 2000; Shaywitz, 1998). In Finnish, another very transparent orthography, the prevalence rate for adult speakers was estimated at around 6% (Lyytinen, Leinonen, Nikula, Aro, & Leiwo, 1995). It is important to bear in mind that the children that were labeled as being at risk of dyslexia in our sample may indeed have been behind in reading, decoding, and/or spelling, but may not necessarily be dyslexic or develop dyslexia in the future. We base our criteria solely on the IDA’s definition of a dyslexia diagnosis, which does not necessarily require an explanation to be provided for the actual reading and spelling problems experienced. The reading-related cognitive tasks (i.e., phoneme deletion, RAN, digit span, and verbal fluency) can therefore provide valuable information to support the diagnostic process as they can help establish whether the child indeed has a dyslexia-specific cognitive profile, which, in turn, might explain the literacy problems when they are unexpected in relation to the student’s other cognitive and learning abilities (similar to the Dutch Protocol for Diagnostics and Treatment of Dyslexia, Nationaal Referentiecentrum Dyslexie, 2013). Moreover, we have found in the regression analysis that the automaticity component (component 1) significantly predicts reading in grade 1 and reading and decoding in grade 2. This is an indication that automaticity or naming speed as with the task we used, has an impact on reading competence for early readers of SI.

We hope that with this comprehensive first study we have provided a starting point for future dyslexia research in Indonesia. Of course, our study is limited in that the sample consisted of students from one elementary school based in one of Indonesia’s many cities, while there are many other ethnic groups in other regions that do not speak SI as their first language. As such, the scores we acquired and the norm scores we computed may not be representative of young learners of SI in other regions. Additionally, the size of the sample is also limited to the school investigated. To collect more and more general normative data, the test battery needs to be applied in other parts of the country and other ethnic groups of different socio-economic backgrounds. If these evaluations were to generate inconsistent results, the test battery would require modification. Moreover, it would be recommended to further investigate other factors that could have contributed to the lower scores attained by the at-risk children, such as SES, parents’ educational levels, and school attendance of the child. Given the strong indications of a genetic component in dyslexia, additional data on reading and spelling difficulties among parents and siblings may also provide valuable information. Nonetheless, the overall profile of test scores we obtained suggests that the tests yield valid measures of reading acquisition and dyslexia, while the phoneme deletion and RAN tasks as well as the spelling and OCT tests appear to provide useful information to better interpret the reading and decoding fluency outcomes. However, as mentioned before, the difficulty level of the latter two tests may need to be adjusted for implementation in higher grades. It would also be interesting to see whether the addition of a silent reading test (assessing silent reading fluency) would generate further useful information. Finally, we recommend to always include both individual teacher ratings and school grades in the final diagnosis as our results showed significant differences between the typical and the at-risk-of-dyslexia readers on both variables that supported the test results of the reading assessment battery.

## Appendix 1

See Table 7.

**Table 7:** Grade 1 factor analyses using varimax rotation

	Component		
	1	2	3
Writing		0.770	
OCT		0.709	
TTR		0.569	
Digit span-F		0.360	0.719
Verbal fluency		0.397	−0.663
Phoneme deletion		0.624	
RAN digits	0.905		
RAN letters	0.796		
RAN colors	0.882		

Reading and decoding fluency are excluded for regression analysis

Factor loadings <0.30 are suppressed

## Appendix 2

See Table 8.

**Table 8:** Grade 2 factor analyses using varimax rotation

	Component		
	1	2	3
Writing			0.784
OCT		0.439	0.693
TTR	0.573	0.324	
Digit span-F		0.645	−0.432
Verbal fluency		0.520	
Phoneme deletion		0.803	
RAN digits	0.896		
RAN letters	0.727		0.361
RAN colors	0.815		

Reading and decoding fluency are excluded for regression analysis

Factor loadings <0.30 are suppressed
